# Seminal Plasma and Seminal Plasma Exosomes of Aged Male Mice Affect Early Embryo Implantation *via* Immunomodulation

**DOI:** 10.3389/fimmu.2021.723409

**Published:** 2021-10-12

**Authors:** Dandan Wang, Kadiliya Jueraitetibaike, Ting Tang, Yanbo Wang, Jun Jing, Tongmin Xue, Jinzhao Ma, Siyuan Cao, Ying Lin, Xiaoyan Li, Rujun Ma, Xi Chen, Bing Yao

**Affiliations:** ^1^ Department of Reproductive Medicine, Affiliated Jinling Hospital, Medicine School of Nanjing University, Nanjing, China; ^2^ Department of Reproductive Medicine, Affiliated Jinling Hospital, Nanjing Medical University, Nanjing, China; ^3^ School of Life Sciences, Nanjing University, Nanjing, China

**Keywords:** seminal plasma, seminal plasma exosomes, dendritic cells, advanced-age male fertility, uterine immune microenvironment, embryo implantation

## Abstract

Seminal plasma (SP), particularly SP exosomes (sExos), alters with age and can affect female mouse uterine immune microenvironment. However, the relationship between fertility decline in reproductively older males, and SP and sExos age-related changes, which may compromise the uterine immune microenvironment, remains unclear. The present study demonstrated that the implantation rate of female mice treated with SP from reproductively older male mice (aged-SP group) was lower than that of those treated with SP from younger male mice (young-SP group). RNA-sequencing analysis revealed altered levels of dendritic cell (DC)-related cytokines and chemokines in the uteri of the former group compared with those of the latter group. *In vivo* and *in vitro* experiments demonstrated a weaker inhibitory effect of aged SP on DC maturation than of young SP upon stimulation. After isolating and characterizing sExos from young and advanced-age male mice, we discovered that insemination of a subset of the aged-SP group with sExos from young male mice partially recovered the implantation rate decline. Additional *in vivo* and *in vitro* experiments revealed that sExos extracted from age male mice exerted a similar effect on DC maturation as SP of aged mice, indicating an age-related sExos inhibitory effect. In conclusion, our study demonstrated that age-related alterations of sExos may be partially responsible for lower implantation rates in the aged-SP group compared with those in the young-SP group, which were mediated by uterine immunomodulation. These findings provide new insights for clinical seminal adjuvant therapy.

## Introduction

Due to social pressure, extension of life expectancy, popularization of effective contraception, and the development of assisted reproductive technology, delayed childbirth has become extremely common, highlighting the issue of advanced-age-related fertility decline. Advanced paternal age, which refers to paternal age over 40, are known to significantly impact fertility and offspring health (1), with male age negatively correlated with semen volume, sperm motility, sperm morphology, reproductive hormone levels, testicular function, chromosome structure, and sperm DNA integrity (2–4). Irreversible cell damage and senescence from age-related inflammation and macromolecular dysfunction (5) produce a senescence-associated secretory phenotype (SASP) characterized by the increased release of pro-inflammatory cytokines, chemokines, and tissue-destructive proteases (6). Increased extracellular vesicle (EV) release has been reported in senescent cells, compared with normal cells, indicating an important EV role in SASP development (7). EVs can be largely divided into three groups, among which the smallest EV exosomes (Exos) varying between 0.03 and 0.15 μm are the focus of the attention of most studies in biomedicine, since they regulate multiple intercellular signaling in physiological and pathological situations (8, 9).

Seminal plasma (SP), an important component of male body fluid, is not only a vehicle for carrying spermatozoa to fertilize oocytes but also an essential actor in female immune response regulation and embryo implantation (10–12). Studies have demonstrated that semen can cause an inflammatory response in human female reproductive mucosa, including the production of inflammatory cytokines and chemokines (13–15), upregulation of cyclooxygenase-2 (16), and recruitment of dendritic cells (DCs) and cervical infiltrating neutrophils (14, 17, 18). Interestingly, recent studies have reported that SP may be beneficial to implantation by promoting DC tolerance (19). Tolerant DCs are more resistant to maturation upon various stimulations. Yasuda et al. discovered that immature phenotype made up the main component of uterine DCs (uDCs) 3.5 days post coitum (dpc), indicating that immature DCs may play an important role in successful implantation (20) and demonstrating the influence of male SP on the female reproductive immune microenvironment.

A typical mammalian ejaculation contains trillions of EVs, a primary component of SP. SP exosomes (sExos) and SP provide immunomodulatory functions in the uterus and may be involved in embryo implantation (21).

Based on these previous findings, we hypothesized that during the aging process of advanced-age males, production of senescence-related SP and sExos alters the uterine immune microenvironment, thus affecting embryo implantation. Our research elucidates the influence of SP and sExos from advanced-age male mice on embryo implantation, demonstrating age-related male fertility decline.

## Materials and Methods

### Mice

Specific pathogen-free (SPF) grade 6- to 8-week-, 12- to 14-week-, and 12- to 18-month-old male and 5- to 6-week-old female C57BL/6 mice and 6- to 8-week-old female Institute of Cancer Research (ICR) mice were purchased from Nanjing Medical University Laboratory Animal Co., Ltd. (Certificate number SCXK (Su) 2016-0002). The mice were housed in 12/12-h light/dark cycle, with unrestricted access to food and water. All animal experiments were approved by the Animal Protection and Use Committee of Jinling Hospital and carried out in accordance with institutional guidelines.

### Animal Experiment Design

As previously described (22), vasectomies were performed on 12- to 14-week-old (young) and 12- to 18-month-old (aged) male C57BL/6 mice, which were ready for mating, to confirm sterility 2 weeks post-surgery. Estrous cycle smears were used to identify estrus 6- to 8-week-old female ICR mice based on vaginal cytology (23), which were divided into two groups: one (young-SP group) caged with young sterilized male mice and the other (aged-SP group) with aged sterilized male mice. Mating was determined by the presence of a copulatory plug, which was checked at 8:00 a.m. daily, and the day of plug detection was defined 0.5 dpc.

### Zygote Collection and Culture

Zygote collection and culture experiments were performed as previously described (24). Briefly, after 46–48 h of 10 IU pregnant mare serum gonadotropin (PMSG) injection, 6- to 8-week-old female ICR mice were injected with human chorionic gonadotropin (HCG) and immediately mated with male mice. Eighteen hours later, the zygotes were harvested and cultured in Quinn’s 1026 cleavage medium (CooperSurgical, Trumbull, CT, USA), supplemented with 10% Quinn’s 3001 serum protein substitute (CooperSurgical) and paraffin oil, at 37°C under 5% CO_2_. The embryos were transferred at the four-cell phase and cultured in Quinn’s 1029 blastocyst medium (CooperSurgical), supplemented with 10% Quinn’s 3001 serum protein substitute (CooperSurgical), to blastocyst stage for subsequent transfers.

### Seminal Plasma Exosomes Administration and Blastocyst Transfer

Copulatory plugs were removed immediately after being detected. For sExos administration, transvaginal injection of 20 μl sExos/mouse was performed at 0.5 dpc or on the morning of estrus day. Briefly, a pipette tip was gently inserted about 5 mm deep into the vaginal lumen of mouse, and 20 μl of sExos was then deposited. For embryo implantation analysis, 16 blastocysts were transferred to each pseudo-pregnant mouse at 3.0 dpc following protocols provided by Pablo Bermejo-Alvarez (22) with little modifications, and mice were euthanized *via* cervical dislocation at 6.5 dpc to analyze implantation rates.

### Mouse Uterus RNA Sequencing and Functional Analysis

TRIzol was used to extract total RNA from the uteri of young-SP and aged-SP group female mice at 3.0 dpc, during the pre-implantation window and prior to embryo transfer. RNA integrity and concentration were assessed using the RNA Nano 6000 Assay Kit of the Bioanalyzer 2100 system (Agilent Technologies, Santa Clara, CA, USA). RNA integrity number (RIN) ≥8 was used as a cutoff for RNA integrity. Sequencing libraries were generated using NEBNext^®^ Ultra™ RNA Library Prep Kit for Illumina^®^ (#E7530L, New England BioLabs, Ipswich, MA, USA) following the manufacturer’s recommendations. High-throughput sequencing was performed on the Illumina HiSeq 2500 system (Illumina, San Diego, CA, USA) at Annoroad Gene Technology (Beijing, China; http://www.annoroad.com). The original data were filtered and aligned to the reference genome using HISAT2 v2.1.0. Fragments per kilobase of transcript, per million mapped reads were calculated to estimate gene expression levels in each sample. DESeq2 was used to determine differential expression between samples. Genes with q < 0.05 and log2 FC > 1 were identified as differentially expressed genes (DEGs). The Kyoto Encyclopedia of Genes and Genomes (KEGG) enrichments of the DEGs were used to determine their molecular interactions and reaction networks. The p-value ≤0.05 and false discovery rate (FDR) q-value ≤0.05 was considered as statistically significant.

### RNA Extraction and Quantitative RT-PCR

Total RNA was extracted from the uteri of young-SP and aged-SP group female mice at 3.0 dpc using a Total RNA Isolation Kit (BEI-BEI Biotech, Zhengzhou, China). The RNA extracted from the uteri of unmated mice between 1:00 p.m. and 4:00 p.m. on the day of estrus was regarded as control. To estimate RNA quality, the ratio of the absorbance contributed by the nucleic acid to the absorbance of the contaminants was calculated and requirements for A_260_/A_280_ ratios are 1.8–2.2. HisScript III RT SuperMix for qPCR (Vazyme Biotech, Nanjing, China) was used for RT-PCR. AceQ qPCR SYBR Green Master Mix (Vazyme Biotech) was used for qPCR, according to the manufacturer’s instructions. The samples were amplified and monitored using a Roche LightCycler 96 Real-time PCR system (Roche Diagnostics, Basel, Switzerland). The fold change in gene expression was calculated using the 2^−∆∆Cq^ method with the housekeeping gene GAPDH as the internal control, which was consistently expressed across samples. Primer-Blast was used to design primers. The primer sequences are listed in **Table 1**. Briefly, primers should have the following properties to ensure the efficiency and specificity: length of 18–24 bases; 40%–60% G/C content; start and end with 1–2 G/C pairs; and melting temperature (Tm) of 50°C–60°C. Primer pairs should have a Tm within 5°C of each other; primer pairs should not have complementary regions.

**Table 1:** Sequence of primers used for real-time quantitative PCR.

Gene name	5′-Sequence-3′
m-Cxcl1-F	CTGGGATTCACCTCAAGAACATC
m-Cxcl1-R	CAGGGTCAAGGCAAGCCTC
m-IL20rb-F	ACCCCTTTAACCGAAATGCAA
m-IL20rb-R	CCTCCAGTAGACCACAAGGAA
m-Fas-F	TATCAAGGAGGCCCATTTTGC
m-Fas-R	TGTTTCCACTTCTAAACCATGCT
m-Cxcl16-F	CCTTGTCTCTTGCGTTCTTCC
m-Cxcl16-R	TCCAAAGTACCCTGCGGTATC
m-Tnfsf10-F	ATGGTGATTTGCATAGTGCTCC
m-Tnfsf10-R	GCAAGCAGGGTCTGTTCAAGA
m-Cx3cl1-F	ACGAAATGCGAAATCATGTGC
m-Cx3cl1-R	CTGTGTCGTCTCCAGGACAA
m-Eda-R	TCAGGGGACTCTGCCACTC
m-Eda-F	CAGGCTGGGCTTTCCAACT
m-Ackr4-F	AGCCAGTACGAAGTGATCTGC
m-Ackr4-R	CTGCGAGCCCAGTGACAAA
m-Il17rb-F	GGCTGCCTAAACCACGTAATG
m-Il17rb-R	CCCGTTGAATGAGAATCGTGT
m-GAPDH-F	TCTTGCTCAGTGTCCTTGC
m-GAPDH-R	CTTTGTCAAGCTCATTTCCTGG

### Uterine Immunohistology and Luminal Fluid Collection

DCs were detected in paraffin-embedded uterine tissues of estrus control, young-SP group, aged-SP group, young-sExos group (perfused with sExos extracted from young male mice), and aged-sExos group (perfused with sExos extracted from aged male mice) females. Intrauterine injection of 10 μg of lipopolysaccharide (LPS; Solarbio, Beijing, China) in a total volume of 25 μl were administered at 0.5 dpc, and uterine luminal fluid was flushed with 50 µl of phosphate-buffered saline (PBS) prior to harvesting uterine tissues at 3.0 dpc. Insoluble material was pelleted at 14,000 × *g* for 10 min, and the supernatant was stored at −80°C until analysis. Uterine tissues were fixed with 4% paraformaldehyde (PFA) in PBS overnight at 4°C and dehydrated in ethanol before paraffin embedding. Tissue sections (3 µM) were cut on a LEiCA RM2016 Rotary Microtome (LEiCA, Shanghai), dewaxed in xylene, and rehydrated. After antigen retrieval process, tissues were surrounded with a hydrophobic barrier using a barrier pen. Non-specific staining between the primary antibodies and the tissue was blocked by incubation in blocking buffer (15% normal rabbit serum (vol/vol) in PBS) at room temperature. Sections were washed in PBS before incubation with anti-CD11c Rabbit pAb (1:100, Servicebio, Wuhan, China) for 1 h. Following washing in PBS, sections were incubated with TRITC-conjugated goat anti-rabbit IgG (1:100, Abcam, Cambridge, UK) overnight at 4°C in a humidified chamber. Sections were washed in PBS before incubation overnight with one of the followings: anti-mouse CD80 fluorescein isothiocyanate (FITC) (1:100, BioLegend, San Diego, CA, USA), anti-mouse CD83 APC (1:100, BioLegend), anti-mouse CD86 FITC (1:100, BioLegend), and anti-mouse MHCII Alexa Fluor^®^ 647 (1:100, BioLegend). Following washing in PBS, DAPI was added, and tissues were incubated for 10 min at room temperature. After rinsing with PBS, tissues were mounted with an anti-fade mounting media. Images were captured using an Eclipse Ci-E microscope (Nikon Instrument, Netherlands). To clearly visualize double-positive cells, the green and red channels were merged using the “AND” gate function in the Image Calculator of ImageJ.

### Preparation of Mouse Seminal Plasma

For collecting young SP and aged SP, three 12- to 14-week-old male C57BL/6 mice and three 12- to 18-month-old male C57BL/6 mice were used, respectively. After being euthanasia, mouse epididymal head, caudal epididymis, prostates, and seminal vesicles were removed from the opened abdominal cavity with microscopic ophthalmic instruments under an anatomical microscope and placed into 600 µl of precooled 0.5% PBS solution. These tissues were cut into small pieces with ophthalmic scissors, and the seminal vesicle fluid was extruded with a sterile syringe tip. The tissue suspension was transferred to a 1.5-ml centrifuge tube and centrifuged at 9,000 × *g* at 4°C for 30 min. The supernatants were collected as young SP and aged SP, respectively, both of which were stored at −80°C before use.

### Enzyme-Linked Immunosorbent Assay

The production of interleukin (IL)-12p70, tumor necrosis factor-α (TNF-α), IL-1β, IL-6, IL-10, and transforming growth factor-beta (TGF-β) was measured by ELISA kits (Enzyme-linked Biotechnology, Shanghai, China). Briefly, uterine luminal fluid or cell culture supernatant was added to the reaction buffer containing 50 μl of horseradish peroxidase (HRP)-conjugated reagent, and the wells were sealed and placed in a 37°C water bath for 60 min. Then, the wells were washed, and solutions A and B were added and incubated for another 15 min. The reaction was stopped by adding the termination buffer. The optical density was detected with a Multimode Microplate Reader (BioTek, Winooski, VT, USA). The concentration of each sample was calculated from the standard curve. The limit of these ELISA kits are as follows: mouse IL-12P70: 31.25–1,000 pg/ml; mouse TGF-β: 25–800 pg/ml; mouse IL-10: 12.5–400 pg/ml; mouse IL-8: 3.75–120 pg/ml; mouse IL-6: 3.75–120 pg/ml; mouse IL-1β: 31.25–1,000 pg/ml; and mouse TNF-α: 20–640 pg/ml.

### Preparation of Dendritic Cells

As previously described (25), bone marrow from the tibia and femur of euthanized C57BL/6 mice were extracted into a culture dish containing Roswell Park Memorial Institute (RPMI) 1640. The bone marrow cell suspension was centrifuged at 1,000 × *g* for 10 min and subsequently, after the addition of 3 ml of red blood cell lysate, at 1,000 × *g* for 5 min. The cells were cultured in the absence or presence of aged SP (dilution of 1:100), young SP (dilution of 1:100), aged sExos, or young sExos in 6-well plates at 1 × 10^6^ cells per well; and 200 ng/ml of granulocyte-macrophage colony-stimulating factor (GM-CSF) and 200 ng/ml of IL-4 were added. Half of the cell culture medium was refreshed every 3 days. On day 6, non-adherent cells in the culture supernatant and loosely adherent cells harvested by gentle washing with PBS were pooled and used as the starting source of material for most experiments [bone marrow-derived DCs (BMDCs)].

### Flow Cytometry and Dendritic Cell Maturation Analysis

BMDCs were stimulated with 1 μg/ml of LPS. After a 24-h culture, the cells were then collected by centrifugation and resuspended in single-cell PBS suspensions. Single-cell suspensions with final cell concentration 1 × 10^6^ cells/ml were blocked with 0.5% bovine serum albumin (BSA) at 4°C for 15 min and incubated with fluorescently labeled antibodies for 30 min. The following antibodies were used for flow cytometry analysis: anti-mouse CD11c PE (0.5 µg/test, BioLegend), anti-mouse CD80 FITC (0.125 µg/test, BioLegend), anti-mouse CD83 APC (0.5 µg/test, BioLegend), anti-mouse CD86 FITC (0.125 µg/test, BioLegend), and anti-mouse MHCII Alexa Fluor^®^ 647 (0.25 µg/test, BioLegend). The cells were subsequently washed, suspended in PBS solution, and analyzed using a BD FACSCalibur flow cytometer (BD Biosciences, Franklin Lakes, NJ, USA). The data were analyzed using FlowJo 7.6.1 software (Tree Star, Inc., Ashland, OR, USA).

### Isolation and Characterization of Seminal Plasma Exosomes

The previously collected SP were each diluted with PBS to a total volume of 10 ml and centrifuged at 300 × *g* for 5 min. The supernatant was transferred to a new centrifuge tube and centrifuged at 3,000 × *g* for 10 min to remove cells or other debris and at 10,000 × *g* for 25 min to remove other larger vesicles. The resulting supernatant was then transferred to a matching overspeed centrifuge tube and centrifuged at 110,000 × *g* for 70 min. After the supernatant was transferred into a new tube, 60 µl of PBS was added to resuspend the pellet, which was then filtered with 0.45- and 0.22-μm filters and collected as young sExos and aged sExos, respectively. All centrifugations were carried out under 4°C, and the sExos were stored under −80°C for at most 1 week before use. Epididymosomes were collected following the same procedure as sExos isolation with only epididymal head, and caudal epididymis was used. The presence and purity of sExos were confirmed using H7700 transmission electron microscopy (Hitachi High-Tech, Tokyo, Japan). The sExos size was directly tracked using the Nanosight NS300 system (NanoSight Technology, Malvern, UK). Samples were manually injected into the sample chamber at ambient temperature. Each sample was measured thrice, and the average value was calculated. Finally, data were analyzed using the nanoparticle tracking analysis (NTA) analytical software, version 2.3.

### Western Blotting of Seminal Plasma Exosomes

Western blotting was used to identify sExos. Total protein was extracted using a radioimmunoprecipitation assay (RIPA) buffer, and protein samples were fractionated by sodium dodecyl sulfate–polyacrylamide gel electrophoresis (SDS-PAGE), analyzed by Western blotting, and visualized. Membranes were blocked with Albumin Bovine (BSA; Biofroxx, Einhausen, Germany) in TBST (10 mM of Tris, pH 7.5, 150 mM of NaCl, and 0.1% Tween-20) and then probed with primary antibodies overnight at 4°C. The following primary antibodies were used: monoclonal rabbit anti-mouse CD63 antibody (1:2,000, Abcam), monoclonal rabbit anti-mouse TSG101 antibody (1:2,000, Santa Cruz Biotechnology, Dallas, TX, USA), polyclonal CD9 antibody (1:1,000, MultiSciences Biotech, Zhejiang, China), monoclonal rabbit anti-mouse Albumin antibody (1:2,000, Abcam), and monoclonal GAPDH antibody (1:5,000, KangChen Biotech, Shanghai, China). After multiple washes in TBST and incubation with HRP-conjugated secondary antibodies (1:20,000, Beyotime, Shanghai, China), the protein bands were visualized using an automatic chemiluminescence image analysis system (Tanon Science & Technology, Shanghai, China). The intensities of protein bands were quantitated using ImageJ Gel Analysis program.

### Tracing of PKH67-Labeled Seminal Plasma Exosomes in the Uterus

PKH67 Green Fluorescent Cell Linker Kits (Sigma-Aldrich) were used to label sExos lipid bilayers. According to the reagent manufacturer’s instructions, 100 μg of freshly isolated sExos was diluted with 500 µl of diluent C and mixed with 500 µl of diluent C, which contained 4 µl of PKH67. After being incubated at room temperature for 5 min, 500 µl of Exosome-depleted FBS Media Supplement (System Biosciences, Palo Alto, USA) was added to halt the staining. The PKH67-labeled pure sExos were then separated from the unbound dye using the ExoQuick exosome precipitation solution (Bomais, Beijing) and were resuspended with 60 µl of PBS. Unstained sExos were used as a negative control. Each mouse was transvaginally injected with 20 µl of PKH67-labeled sExos or control sExos and euthanized 6 h later. The uteri were removed, frozen, and sectioned. After blocking with 2% BSA, slides were incubated overnight with anti-Vimentin antibody (1:200, Abcam) and anti-Cytokeratin 19 antibody (1:400, Abcam) at 4°C and then with secondary antibodies at room temperature for 1 h. The nuclei were then stained with DAB for 15 min, and images were digitized with an IX73 fluorescence microscope (Olympus Corporation, Shinjuku, Tokyo, Japan).

### Internalization of Seminal Plasma Exosomes by Bone Marrow-Derived Dendritic Cells

sExos were labeled with PKH67 green fluorescent dye and added to BMDCs cultured in glass-bottom chamber slides. After 24 h of incubation, the cells were placed on poly-l-lysine-coated glass coverslips (12 mm) during 20 min at room temperature. Then cells were washed with PBS and fixed in 4% PFA (10 min on ice) and washed twice with 0.1 mM of glycine in PBS. Subsequently, cells were incubated with a mouse anti-CD11c Rabbit pAb (1:100, Servicebio) for 1 h and revealed with TRITC-conjugated goat anti-rabbit IgG (1:100, Abcam) for 45 min. Then BMDCs were stained with Hoechst for 10 min. Coverslips were mounted on glass slides using Fluoromount and visualized with a LSM 710 laser scanning confocal microscope (Carl Zeiss, Oberkochen, Germany).

### Statistical Analysis

GraphPad Prism 8 and SPSS v.20 software packages were used for statistical analysis. Data are expressed as mean ± standard error of the mean. Each experiment was performed in triplicate technical and biological replicates. The differences among different groups and between two groups were assessed by one-way ANOVA with the Holm–Sidak post-hoc test and paired-samples t-test, respectively. For all tests, a bilateral test probability of p < 0.05 was considered to represent a statistically significant difference.

## Results

### Embryo Implantation Rate Reduced in Aged-Seminal Plasma Group

**Figure 1:**
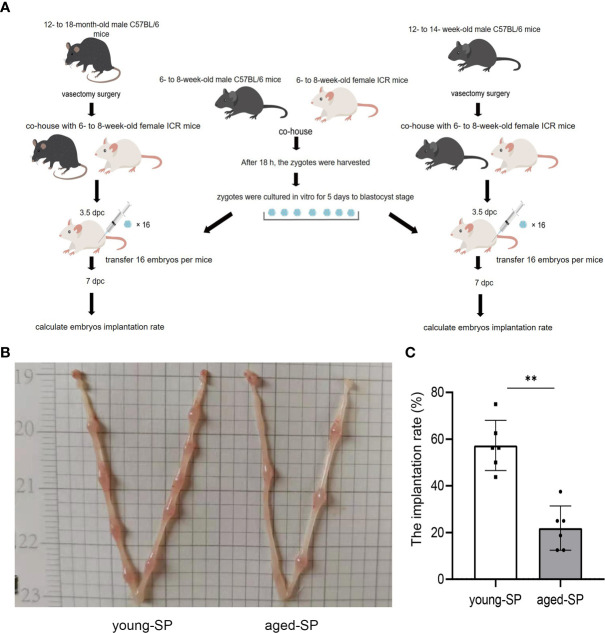
The embryo implantation in the aged-SP group and young-SP group. **(A)** Schematic diagram of experimental design. **(B)** Embryo implantation sites of two groups of recipient female mice on 6.5 dpc. **(C)** Quantitative histogram of embryo implantation rates in two groups of recipient female mice (n = 6). The differences between two groups were assessed by paired-samples t-test. **p < 0.01. The implantation rate (%): the number of implants/number of embryos transferred. SP, seminal plasma; dpc, days post coitum.

In order to determine whether the effects of SP on embryo implantation were related to male age, we constructed two groups of sterilized male models of different age ranges equal to 20–30 and 40–50 years in humans, using embryo transfer to eliminate maternal and gamete factors, as illustrated by the experimental model diagram in **Figure 1A**. Results showed that the implantation rate of the aged-SP group was significantly lower than that of the young-SP group (**Figures 1B, C**), which indicated age-related effects of SP on embryo implantation.

### Uterine Transcriptomics Differed Between Young-Seminal Plasma and Aged-Seminal Plasma Groups

**Figure 2:**
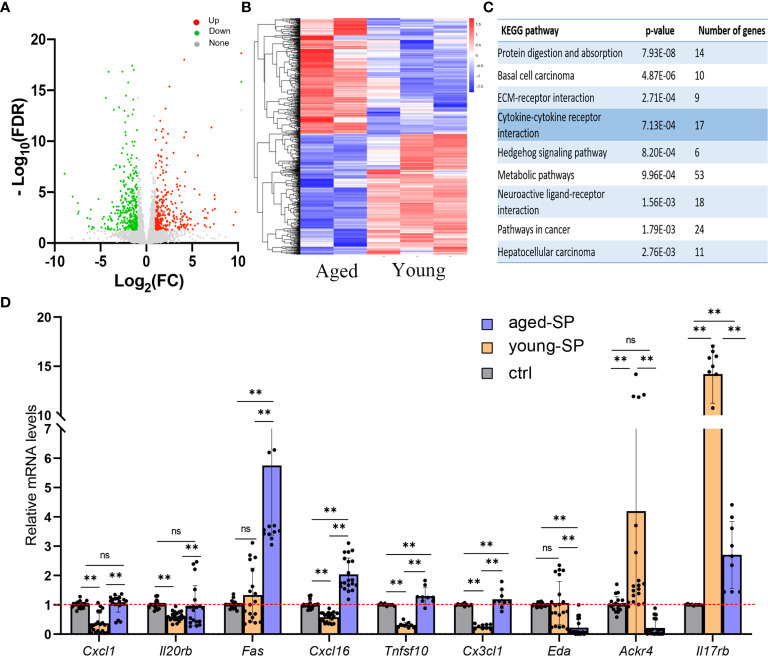
The transcriptomics of the uteri in the young-SP group and aged-SP group. **(A, B)** Volcano map and heatmap depicting clustering and global changes in gene expression for the uterus from aged-SP group and young-SP group as determined by RNA-seq. **(C)** KEGG pathway analysis of transcripts significantly changed. Top nine most highly enriched categories are shown. **(D)** qPCR validation for a subset of genes involved in cytokine–cytokine receptor interaction. *Gapdh* served as an internal control. The differences among different groups were assessed by one-way ANOVA with Holm–Sidak post-hoc test. **p < 0.01. The p-value ≤ 0.05 and FDR q-value ≤ 0.05 were considered as statistically significant. SP, seminal plasma; KEGG, Kyoto Encyclopedia of Genes and Genomes; FDR, false discovery rate; ns, not significant.

To investigate the possible causes of the disparity in implantation rates between the aged-SP and young-SP groups, we analyzed the RNA sequence of 3.0-dpc uteri from both groups. In hierarchical clustering (data not shown), a sample in the young-SP group was identified as an outlier, which laid in its own branch and extended from the very root of the tree. Outliers increase the variability in data, which decreases statistical power. Consequently, we excluded this outlier, and the principal component analysis of five samples finally included is presented in **Supplementary Figure 1**. As gene cluster maps demonstrate, 632 differentially expressed transcripts were identified, among which 296 genes were upregulated and 336 genes were downregulated (**Figures 2A, B**). The results of KEGG pathway analysis based on these genes primarily identified protein digestion and absorption, basal cell carcinoma, ECM–receptor interaction and cytokine–cytokine receptor interaction (**Figure 2C**). Cytokines have been primarily defined as modulators of the immune system, and the balance in their relative abundance has been shown to impact embryo implantation (26). Among 17 genes included in cytokine–cytokine receptor interaction, nine have been previously identified to be involved in DC migration and maturation. Tolerogenic DCs that are resistant to maturation seemed to be involved in the generation of tolerance to the embryos (27). We therefore validated a subset of mRNAs related to DCs in cytokine–cytokine receptor interaction pathway by qRT-PCR (**Figure 2D**). Results from the qPCR analysis of total RNA extracted from 3.0-dpc uteri during the pre-implantation window and prior to embryo transfer identified an increase of *Cxcl1*, *Il20rb*, *Fas*, *Cxcl16*, *Tnfsf10*, and *Cx3cl1* and a decrease of *Eda*, *Ackr4*, and *Il17rb* expression levels in the uteri of the aged-SP group compared with those of the young-SP group, which were inconsistent with the RNA-seq results. When compared with the virgin control, young-SP group and aged-SP group presented different variation trends, which proposed a complex regulation network of SP on uterine immune status. These results indicated that SP from advanced-age males impacts the embryo implantation-related signaling pathways, especially those involving DC migration and maturation, differently than SP from younger males.

### Seminal Plasma Exerted an Inhibitory Effect on Dendritic Cell Maturation, Which Was Attenuated in Advanced Age

**Figure 3:**
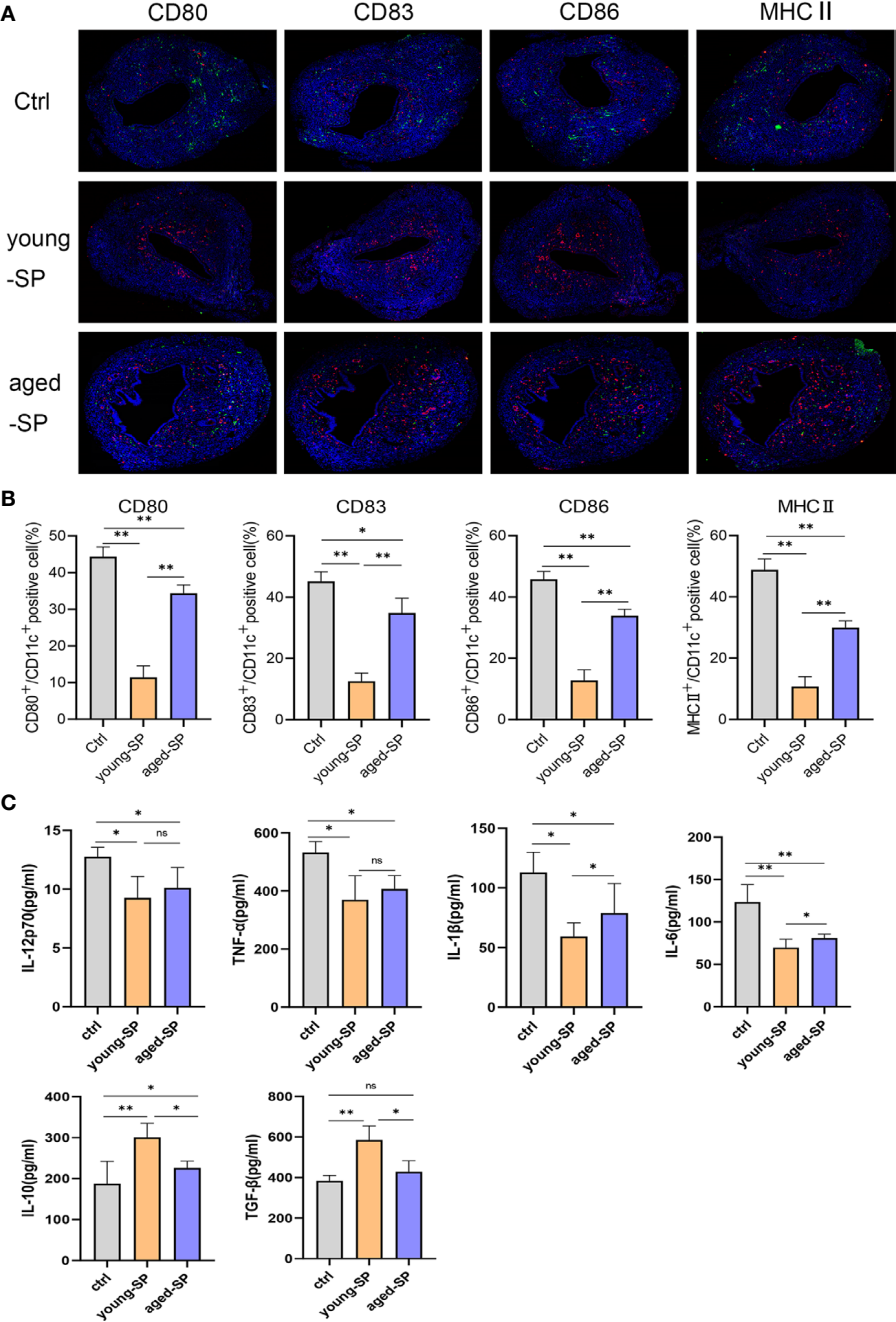
Alterations of uterine DC maturation status between young-SP group and aged-SP group *in vivo*. **(A)** Representative sections of the uteri from control group, young-SP group, and aged-SP group mice, immunostained with anti-CD11c (red) and anti-CD80/CD83/CD86/MHCII (green) antibodies. **(B)** Histomorphometric quantification of DC densities in the myometrium and endometrium of control group, young-SP group, and aged-SP group mice. **(C)** ELISA analysis demonstrating the concentration of IL-12p70, TNF-α, IL-1β, IL-10, TGF-β, and IL-6 in uterine luminal fluid (mean ± SD, n = 6).The differences among different groups were assessed by one-way ANOVA with Holm–Sidak post-hoc test. *p < 0.05 and **p < 0.01. DC, dendritic cell; SP, seminal plasma; ns, not significant.

**Figure 4:**
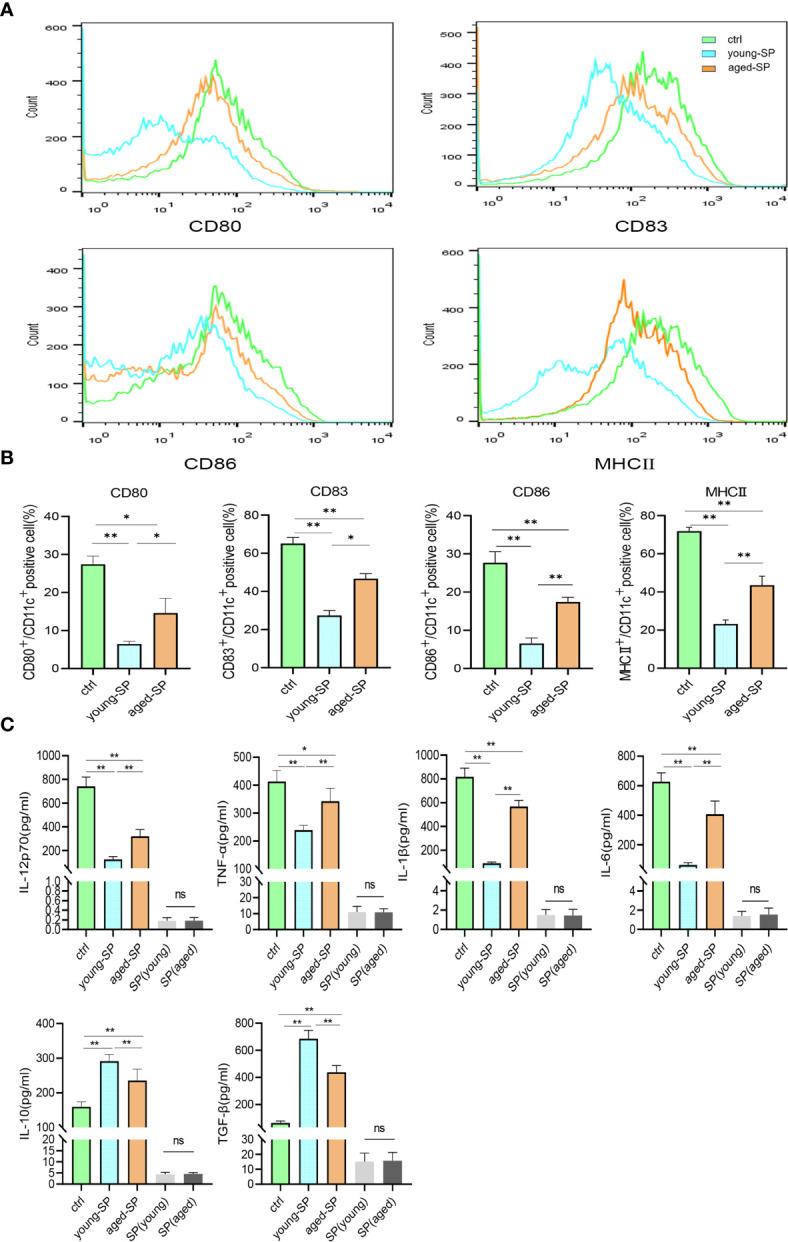
Alterations of BMDC maturation status between young-SP group and aged-SP group *in vitro*. **(A)** Fluorescence-activated cell sorting (FACS) analysis of CD80, CD83, CD86, and MHCII in culture supernatant. The x-axis represents the fluorescence intensity, and the y-axis represents the percentage of positive cells. **(B)** Quantification of CD11c+ CD80+ DC, CD11c+ CD83+ DC, CD11c+ CD86+ DC, CD11c+ MHCII+ DC positive cells as seen in panel A (mean ± SD, n = 6). **(C)** ELISA analysis demonstrating the concentration of IL-12p70, TNF-α, IL-1β, IL-10, TGF-β, and IL-6 in the culture supernatant (mean ± SD, n = 6). The differences among different groups were assessed by one-way ANOVA with Holm–Sidak post-hoc test. *p < 0.05 and **p < 0.01. BMDC, marrow-derived dendritic cell; SP, seminal plasma; DC, dendritic cell; ns, not significant.

To investigate whether DC maturation was involved in the age-related impact of SP on embryo implantation, uterine tissues of young-SP and aged-SP groups were stained with DC maturation markers CD80, CD83, CD86, and MHCII and were visualized. Immunohistology results revealed that both young SP and aged SP could induce DC accumulation in the uteri, while the percentage of mature DCs was higher in aged-SP group than young-SP group (**Figures 3A, B**). Compared with immature phenotype, mature phenotype of DCs produce higher levels of IL-12p70, IL-1b, TNF-a, IL-6, and lower levels of IL-10, TGF-b in response to LPS (19). To further verify whether DC maturation status was altered, uterine luminal fluid in aged-SP and young-SP groups were collected, and ELISA was performed to detect inflammatory cytokines related to DC phenotype (**Figure 3C**). Results showed that the expression profiles of inflammatory cytokines in young-SP group were better aligned with immature DC phenotype than aged-SP group in response to LPS, indicating an inhibitory effect of SP on uterine DC maturation, which was attenuated in the aged-SP group. With emerging evidence showing that SP could impact DC maturation *in vitro*, BMDCs were used as *in vitro* model of DCs to investigate whether a direct impact of SP on DCs existed. CD11c was used to identify BMDCs according to the gating strategy depicted in **Supplementary Figure 2**. Flow cytometry analysis revealed that the expression levels of DC maturation markers CD80, CD83, CD86, and MHCII were higher in the aged-SP group than in the young-SP group (**Figures 4A, B**). In the cell culture supernatant, DC-related inflammatory cytokines presented identical expression profiles as an *in vivo* model (**Figure 4C**). These results indicated that SP exerted an inhibitory effect on DC maturation, which was attenuated in advanced age.

### Characterization of Seminal Plasma Exosomes Extracted From Young and Aged Male Mice

**Figure 5:**
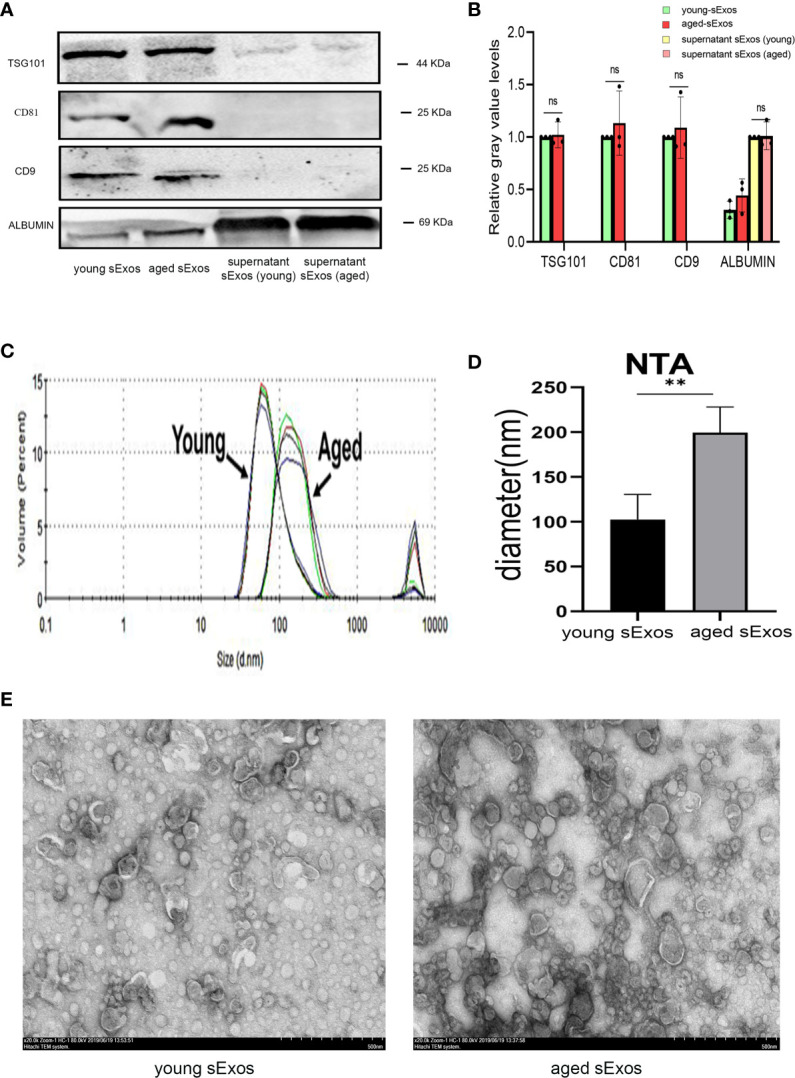
Characterization of sExos extracted from young and aged male mice. **(A)** Western blotting analysis on sExos using antibodies against the positive markers (TSG101, CD81, and CD9) and negative marker Albumin after BCA (Bradford) protein quantification assay. **(B)** Quantification of band intensity as seen in panel **(A, C)** Representative particle size distribution diagrams of sExos from two groups of mice as determined by nanoparticle tracking analysis. **(D)** Quantification of panel C (mean ± SD, n = 6). **(E)** TEM images of sExos isolated from young and aged male mice. Bar = 500 μm. The differences between two groups were assessed by paired-samples t-test. **p < 0.01. sExos, seminal plasma exosomes; BCA, bicinchoninic acid; TEM, transmission electron microscopy; BMDC, marrow-derived dendritic cell; SP, seminal plasma; DC, dendritic cell; ns, not significant.

sExos have been proposed as the main component of SP to provide immunomodulatory functions in the uterus (21). To investigate whether sExos contribute to the phenotype caused by SP, sExos of the young and aged male groups were extracted, and further experiments were performed. Western blotting analysis of sExos extracted from young and aged mice verified the presence of universal exosome markers TSG101 and CD63 (**Supplementary Figure 3A**). Further Western blotting analysis of sExos extracted from a single mouse confirmed minor increase without significance in the abundance of sExos with a similar expression level of sExos-positive markers TSG101, CD81, and CD9 in aged group compared with young group (**Figures 5A, B**). Purity was assessed based on the presence of negative marker Albumin, and the results revealed a small amount of Albumin contamination, which were aligned with other literature (28) (**Figures 5A, B**). NTA revealed that the average sExos size of the young and aged groups is, respectively, 102.8 nm and 206.6 nm (**Figures 5C, D**). Transmission electron microscopy analysis verified the presence of separated or clustered membrane-bound particles in the extracted sExos (**Figure 5E**). These results confirmed successful sExos extraction and an increase in the size of sExos from aged male mice.

### Intrauterine Perfusion With Young Mouse Seminal Plasma Exosomes Partially Rescued the Decline in Aged-Seminal Plasma Group Embryo Implantation Rate

**Figure 6:**
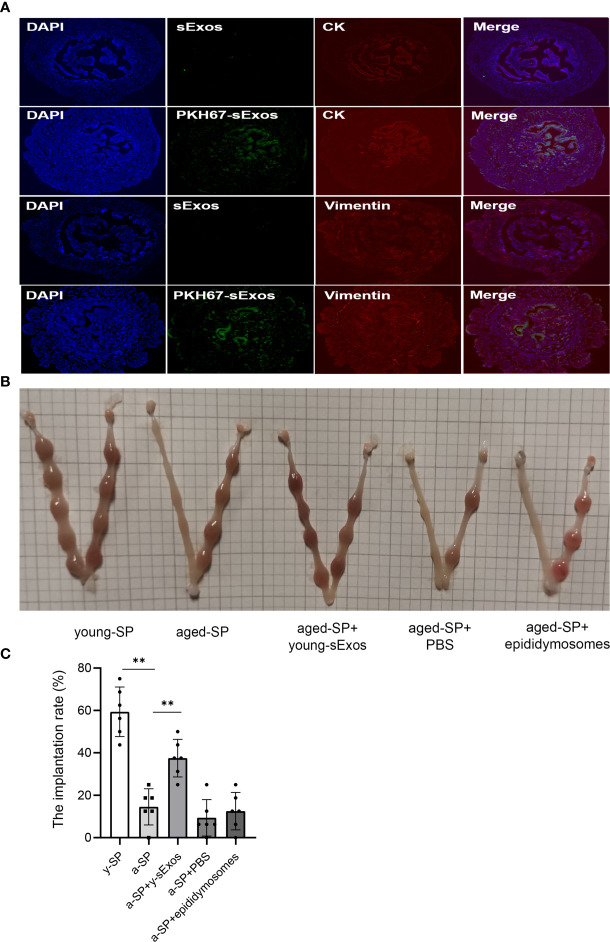
The increase of embryo implantation rate after perfusion with young sExos in the aged-SP group. **(A)** Immunofluorescence staining of the uteri after perfusion with sExos from young male mice. Red fluorescence shows antibody reactivity against Vimentin and Cytokeratin 19, green fluorescence shows PKH67-labeled sExos, and blue fluorescence shows nuclear DAPI reactivity (magnification, ×200). **(B)** Embryo implantation sites of young-SP, aged-SP, aged-SP+young-sExos, aged-SP+PBS, and aged-SP+epididymosomes group female mice on 6.5 dpc. **(C)** Quantitative histogram of the embryo implantation rates in five groups of recipient female mice as seen in panel B (mean ± SD, n = 6).The differences among different groups were assessed by one-way ANOVA with Holm–Sidak post-hoc test. **p < 0.01. sExos, seminal plasma exosomes; SP, seminal plasma; PBS, phosphate-buffered saline; dpc, days post coitum.

To further explore whether young mouse sExos have beneficial effects on the decline of aged-SP group embryo implantation rate, remedial experiments were performed. PKH67 immuno-fluorescence labeling verified sExos entry into the uteri (**Figure 6A**). Further results demonstrated an implantation rate increase in the aged-SP group after perfusion with young sExos (**Figures 6B, C**). Epididymosomes are an important component of the sExos, but in our vasectomized model, epididymosomes would not be present. As such, intrauterine perfusion with epididymosomes extracted from young mice (young epididymosomes) were performed to illustrate whether epididymosomes contributed to the remedial effect of young sExos. Western blotting results confirmed successful extraction of epididymosomes with positive markers CD63, TSG101, and CD9 and negative marker Albumin (**Supplementary Figure 3B**), while no beneficial impact on declined implantation rate in aged-SP group was observed after exerting young epididymosomes (**Figures 6B, C**). These results revealed the beneficial effect of sExos excluding epididymosomes of young subjects on declined embryo implantation rate in aged-SP group.

### Seminal Plasma Exosomes Exerted an Inhibitory Effect on Dendritic Cell Maturation, Which Was Attenuated in Advanced Age

**Figure 7:**
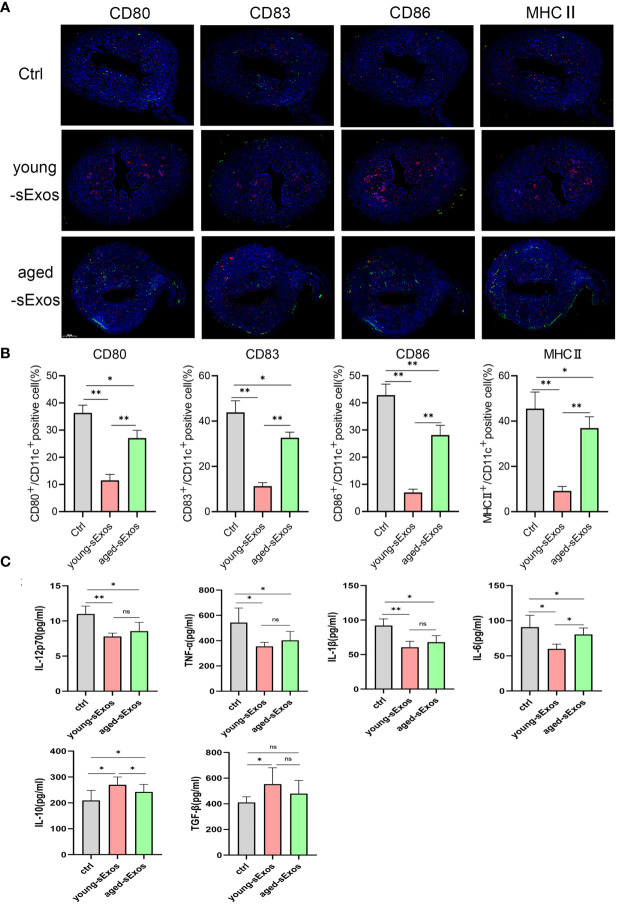
Alterations of uterine DC maturation status between young-sExos group and aged-sExos group *in vivo*. **(A)** Representative sections of the uteri from control group, young-sExos group, and aged-sExos group mice, immunostained with anti-CD11c (red) and anti-CD80/CD83/CD86/MHCII (green) antibodies. **(B)** Histomorphometric quantification of DC densities in the myometrium and endometrium of control group, young-sExos group, and aged-sExos group mice. **(C)** ELISA analysis demonstrating the concentration of IL-12p70, TNF-α, IL-1β, IL-10, TGF-β, and IL-6 in uterine luminal fluid (mean ± SD, n = 6). The differences among different groups were assessed by one-way ANOVA with Holm–Sidak post-hoc test. *p < 0.05 and **p < 0.01. DC, dendritic cell; sExos, seminal plasma exosomes; ns, not significant.

**Figure 8:**
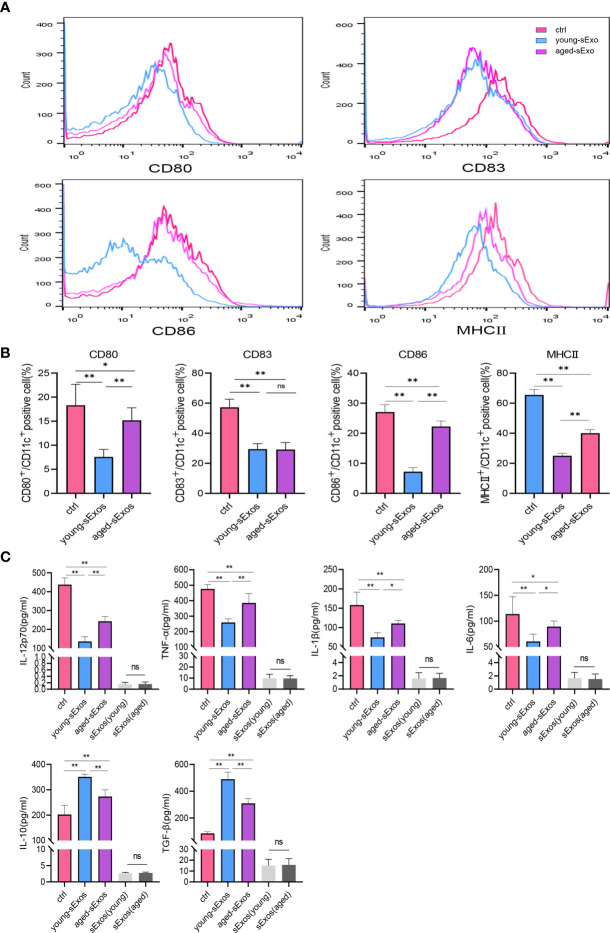
Alterations of BMDC maturation status between young-sExos group and aged-sExos group *in vitro*. **(A)** Fluorescence-activated cell sorting (FACS) analysis of CD80, CD83, CD86, and MHCII in culture supernatant. The x-axis represents the fluorescence intensity, and the y-axis represents the percentage of positive cells. **(B)** Quantification of CD11c+ CD80+ DC, CD11c+ CD83+ DC, CD11c+ CD86+ DC, CD11c+ MHCII+ DC positive cells as seen in panel A (mean ± SD, n = 6). **(C)** ELISA analysis demonstrating the concentration of IL-12p70, TNF-α, IL-1β, IL-10, TGF-β, and IL-6 in the culture supernatant (mean ± SD, n = 6). The differences among different groups were assessed by one-way ANOVA with Holm–Sidak post-hoc test. *p < 0.05 and **p < 0.01. BMDC, marrow-derived dendritic cell; sExos, seminal plasma exosomes; ns, not significant.

For the purpose of investigating whether sExos participate in SP-related alterations of DC maturation, sExos extracted from young and aged male mice were perfused into the uteri of the virgin female mice. Uterine immunohistology with DC maturation markers demonstrated higher percentage of mature DCs after perfusing aged sExos than young sExos (**Figures 7A, B**). Uterine luminal fluid was also collected to detect inflammatory cytokines related to DC phenotype. An increase of IL-6 and a decrease of IL-10 and TGF-β were observed in aged-sExos group compared with young-sExos group (**Figure 7C**). Although no statistic difference was observed regarding IL-12p70, TNF-α, and IL-1β levels, they all increased in aged-sExos group compared with young-sExos group. Further *in vitro* experiments performed to determine whether aged sExos exerted a similar effect on DC maturation as aged SP revealed higher levels of maturation markers CD80, CD86, and MHCII on the surfaces of aged-sExos-treated group DC (**Figures 8A, B**). ELISA analysis of the cell culture supernatant obtained similar results as uterine luminal fluid, which further verified that aged sExos had a weaker inhibitory effect on LPS-induced DC maturation than young sExos (**Figure 8C**). Increased sExos size may be associated with alterations in sExos composition or internalization ability. Regarding the internalization rate by DCs, no significant difference was found between young-sExos and aged-sExos groups (**Supplementary Figure 4**), which indicated an age-related effect of sExos may be associated with alterations in sExos composition. These results showed that aged sExos acted as main components of aged SP in affecting DC maturation.

## Discussion

Function decline in aging semen has been recently identified as another factor besides spermatozoa causing age-related decline in male fertility (29). Emerging evidence has revealed the role of SP in regulating the uterine and fallopian tube environment, thereby enhancing embryo implantation and development (30, 31). Such regulation includes induction of uterine cytokine expression, leading to immune regulation on the uterus of mice (32) and humans (21). The immune cell migration was once believed to be intended for removal of microorganisms and excess sperm (14, 18). However, the SP-induced influx of uterine cytokines and chemokines is now believed to promote maternal tolerance of paternal antigens. T cell-activating macrophages and DCs are recruited into uterine tissue in response to semen-induced cytokine and chemokine signals (33). An article published recently verified migration of uDCs from the periphery just before implantation (20). Their data also showed an increase of immature uDCs on day 3.5 pc, which support the concept that immature DCs may participate in embryo implantation *via* regulating fetal–maternal tolerance (34). Numerous studies have revealed an age-related decline in embryo implantation rate, largely mediated by uterine cytokines and chemokine inducement (15, 29, 35). In our results, we found an age-related effect of SP on embryo implantation, accompanied by alterations in uterine cytokines, which may be responsible for the migration or maturation of DCs (36, 37). GM-CSF-derived BMDCs have been used as a model to study DC biology in countless studies. It has generally been thought that non-DCs can be eliminated by early washing steps, discarding highly adherent cells and enriching or sorting for CD11c+ cells. However, recent studies reckon that CD11c+ fraction of GM-CSF cultures comprises macrophages and DCs (38, 39). In our study, we found alterations in DC maturation markers and changes in cytokines involved in successful implantation, which were not specifically secreted by DCs, with emerging evidences verifying their expressions in both DCs and macrophages (39). Therefore, we assumed that the effects observed after various treatments in BMDCs could be mainly attributed to macrophages and DCs, or even CD11c− cells were included. Although we cannot conclude that alterations were mediated by DCs alone, our data do strongly suggest that DCs are at least largely involved. Tolerogenic DCs seemed to be involved in the generation of tolerance to the embryos (27). A previous study showed that DCs tend to differentiate into tolerogenic DCs, which failed to develop a mature phenotype in response to LPS in the presence of SP (19). Our *in vivo* and *in vitro* results confirmed alterations of DC maturation status between young-SP and aged-SP groups, which indicated that the lower implantation rate observed in the aged-SP group may be associated with the diminished inhibitory effect of aged SP on DC maturation.

Semen contains sperm, testosterone (40), soluble proteins—such as TGF-β and interferon-γ (41)—that can interact with cells in the female reproductive tract, and abundant levels of sExos (42), which are mainly secreted from the prostate, and seminal vesicles (43). We successfully isolated and characterized mouse sExos with the presence of positive markers. It has been demonstrated that senescent cells release more Exos with different compositions, likely contributing to the SASP (44). No significant difference between young and aged groups was observed regarding the quantity of sExos, which indicated that different phenotypes caused by sExos may be more associated with their composition than quantity. NTA analysis revealed that sExos from the advanced-age group exhibited increased size, consistent with senescent mesenchymal stem cells Exos (45). Increased sExos size may be associated with alterations in sExos composition or internalization ability. With no difference observed in the internalization rate, we hypothesized that there might be age-related changes in the sExos composition. Related research has confirmed that sExos can regulate the immune response and gene expression in the female reproductive tract (11, 46, 47), eventually contributing to embryo implantation and gestation. In our results, young sExos partially recovered the declined implantation rate in the aged-SP group, indicating that sExos may contribute to the age-related changes of SP. Zhao et al. demonstrated that sExos secreted by 5-aminolevulinic acid photodynamic therapy-treated squamous carcinoma cells can promote DC maturation to exert an anti-tumor immunity function (48). We found that aged sExos exerted a similar effect on DC maturation to aged SP. The current debate over the clinical benefits of sexual intercourse or intrauterine SP perfusion prior to embryo transfer (49–51) has not considered physiological and pathological conditions such as male age. Uterine response intensity and quality are affected by semen composition, including antigen and immunomodulatory adjuvant content. The alteration of SP components under various physiological and pathological conditions may be an immune-mediated quality control process signal to the female reproductive tract (52), leading to different pregnancy and birth outcomes. SP from advanced-age subjects presented no obvious benefits in our study, suggesting that male age should factor into seminal adjuvant therapy.

In conclusion, our study demonstrates that age-related alterations of sExos may be partially responsible for lower implantation rates in the aged-SP group compared with those in the young-SP group, which were mediated by uterine immune status changes. These findings provide new insight and scientific basis for future clinical seminal adjuvant therapy.

## Data Availability Statement

The RNA sequencing data associated with this study are publicly available in NCBI (GEO) under the accession number GSE180444.

## Ethics Statement

The animal study was reviewed and approved by The Animal Protection and Use Committee of Jinling Hospital.

## Author Contributions

BY and XC: study design and final version of manuscript approval. YW, RM ,YL and XL: data acquisition. JM, TX, SC and JJ: data analysis and interpretation. DW KJ and TT: drafting manuscript, performing experiments and responsible for the integrity of the data analysis. All authors contributed to the article and approved the submitted version.

## Funding

This work was supported by the National Key Research and Development Program of China (2018YFC1004700) and National Natural Science Foundation of China (81971373).

## Conflict of Interest

The authors declare that the research was conducted in the absence of any commercial or financial relationships that could be construed as a potential conflict of interest.

## Publisher’s Note

All claims expressed in this article are solely those of the authors and do not necessarily represent those of their affiliated organizations, or those of the publisher, the editors and the reviewers. Any product that may be evaluated in this article, or claim that may be made by its manufacturer, is not guaranteed or endorsed by the publisher.

## Supplementary Material

Supplementary Figure 1 Principal component analysis of 5 samples finally included in RNAsequence analysis. Red dots represent female mice in aged-SP group, and green dots represent female mice in young-SP group.

Supplementary Figure 2 Gating strategy of BMDCs.

Supplementary Figure 3 Western blotting analysis of sExos and epididymosomes. **(A)** Western blotting analysis on sExos using antibodies against positive exosomal markers (CD63 and TSG101). From left to right: sExos extracted from young male mice; sExos extracted from aged male mice; **(B)** Western blotting analysis on epididymosomes using antibodies against the positive markers (TSG101,CD63 and CD9 ) and negative marker Albumin after BCA (Bradford) protein quantification assay. From left to right: epididymosomes extracted from young male mice, epididymosomes extracted from aged male mice, remaining supernatant after isolating epididymosomes from young male mice, and remaining supernatant after isolating epididymosomes from aged male mice.

Supplementary Figure 4 Internalization of sExos by BMDCs Immunofluorescence staining of BMDCs after cultured with sExos from young male mice **(A)** and aged male mice **(B)**. Red fluorescence shows antibody against CD11c, green fluorescence shows PKH67 labeled sExos and blue fluorescence shows nuclear (magnification, ×40). **(C)** Quantification of PKH67 positive CD11c+ cells. The differences between two groups were assessed by paired samples t-test, *P < 0.05 and **P < 0.01. (n=150).
