# Supplementary figures and images for: Seminal Plasma and Seminal Plasma Exosomes of Aged Male Mice Affect Early Embryo Implantation via Immunomodulation
Source: Front Immunol. 2021 Oct 12;12:723409. doi: 10.3389/fimmu.2021.723409 (PMC8546305; doi:10.3389/fimmu.2021.723409)

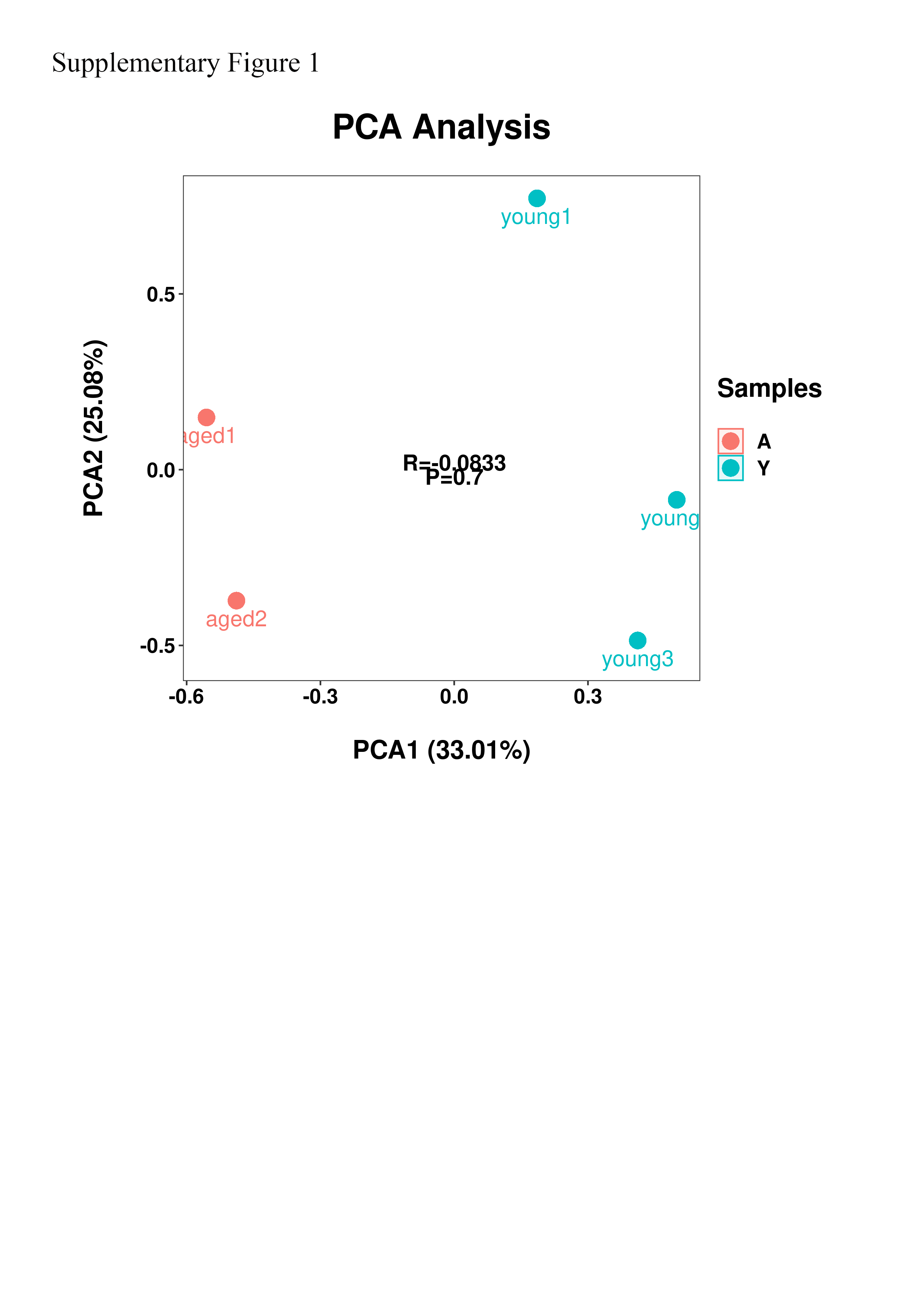

Supplement: Supplementary Figure 1 — Principal component analysis of 5 samples finally included in RNAsequence analysis. Red dots represent female mice in aged-SP group, and green dots represent female mice in young-SP group. [file Image_1.tif]

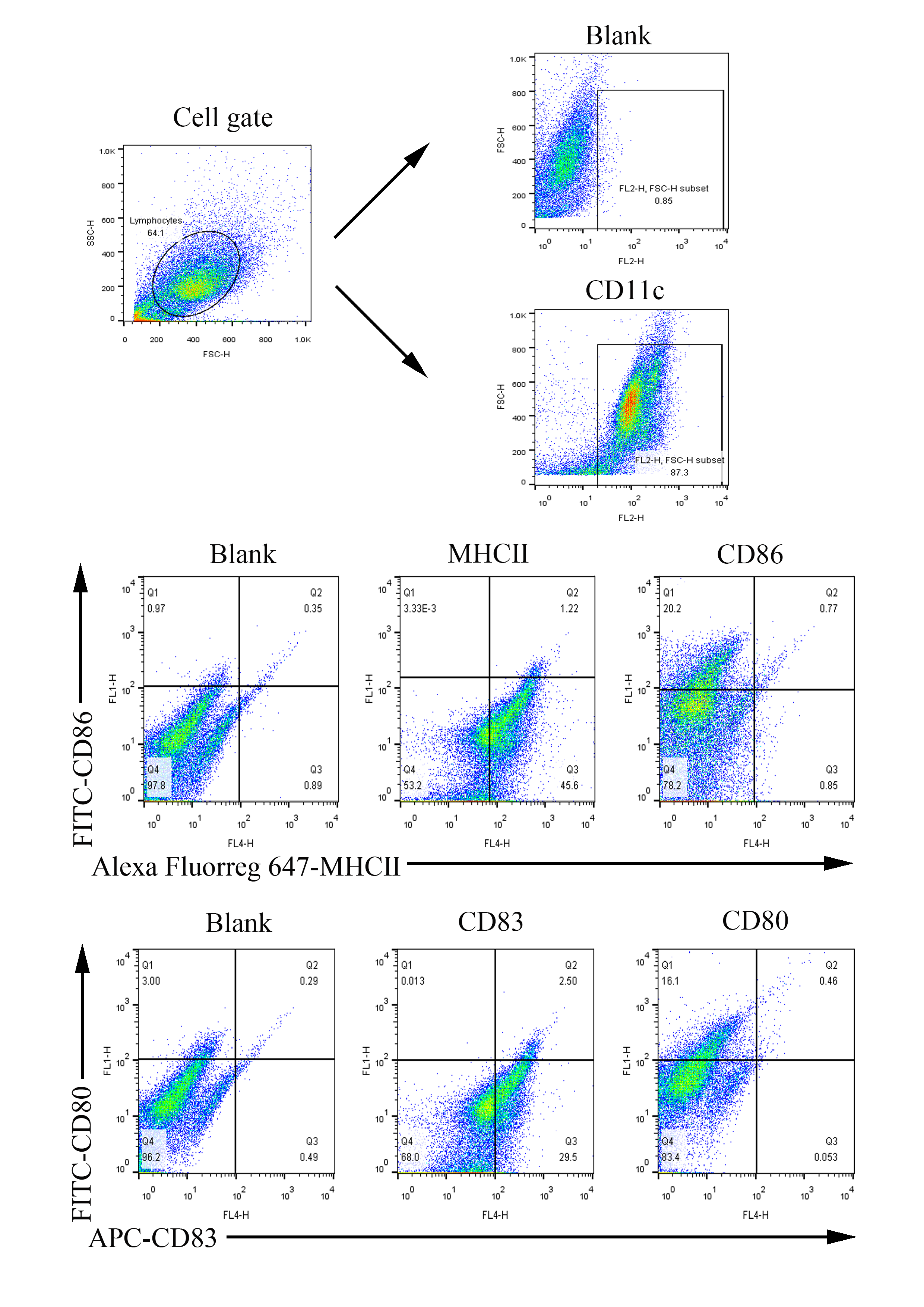

Supplement: Supplementary Figure 2 — Gating strategy of BMDCs. [file Image_2.tif]

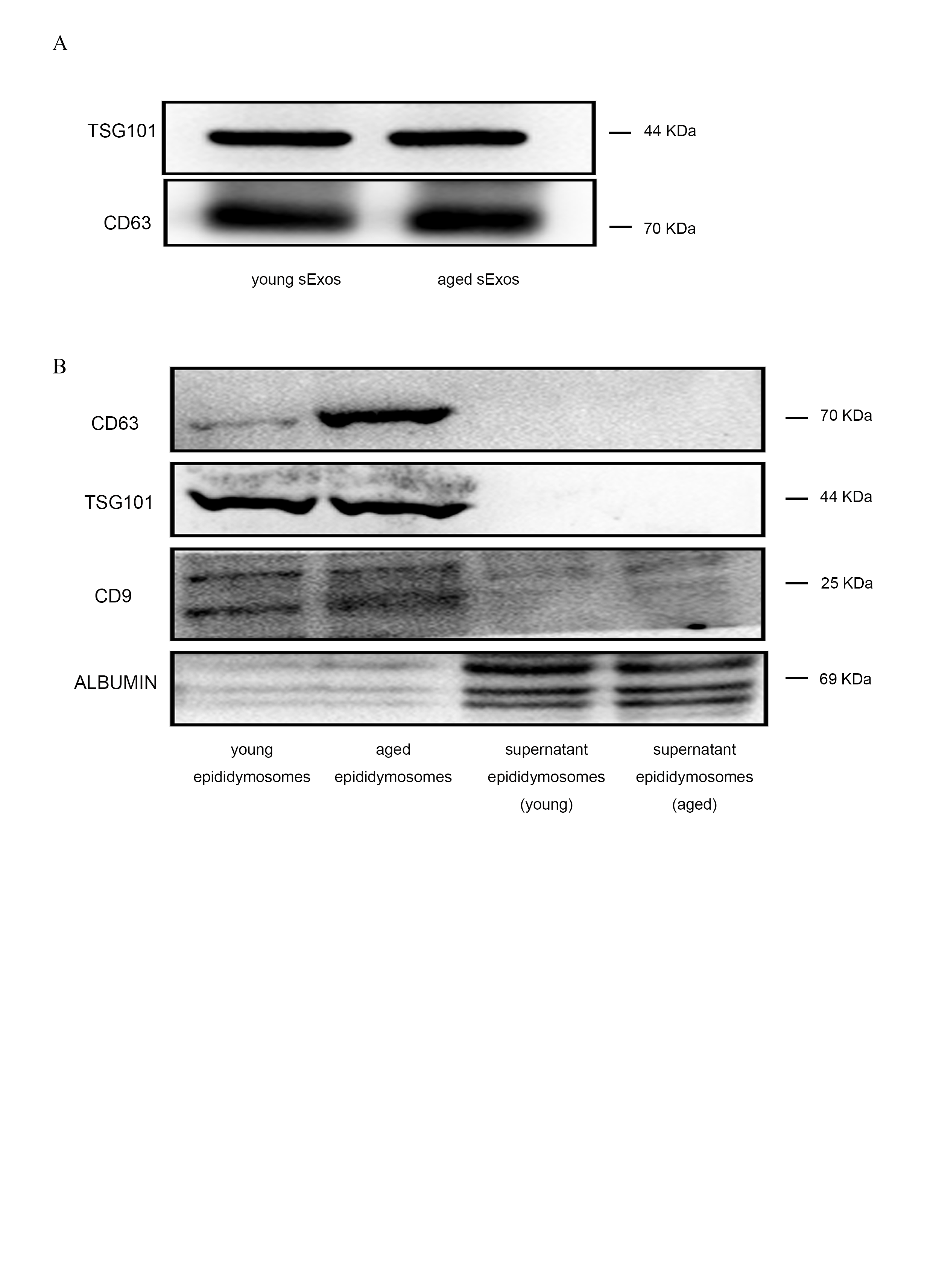

Supplement: Supplementary Figure 3 — Western blotting analysis of sExos and epididymosomes. (A) Western blotting analysis on sExos using antibodies against positive exosomal markers (CD63 and TSG101). From left to right: sExos extracted from young male mice; sExos extracted from aged male mice; (B) Western blotting analysis on epididymosomes using antibodies against the positive markers (TSG101,CD63 and CD9 ) and negative marker Albumin after BCA (Bradford) protein quantification assay. From left to right: epididymosomes extracted from young male mice, epididymosomes extracted from aged male mice, remaining supernatant after isolating epididymosomes from young male mice, and remaining supernatant after isolating epididymosomes from aged male mice. [file Image_3.tif]

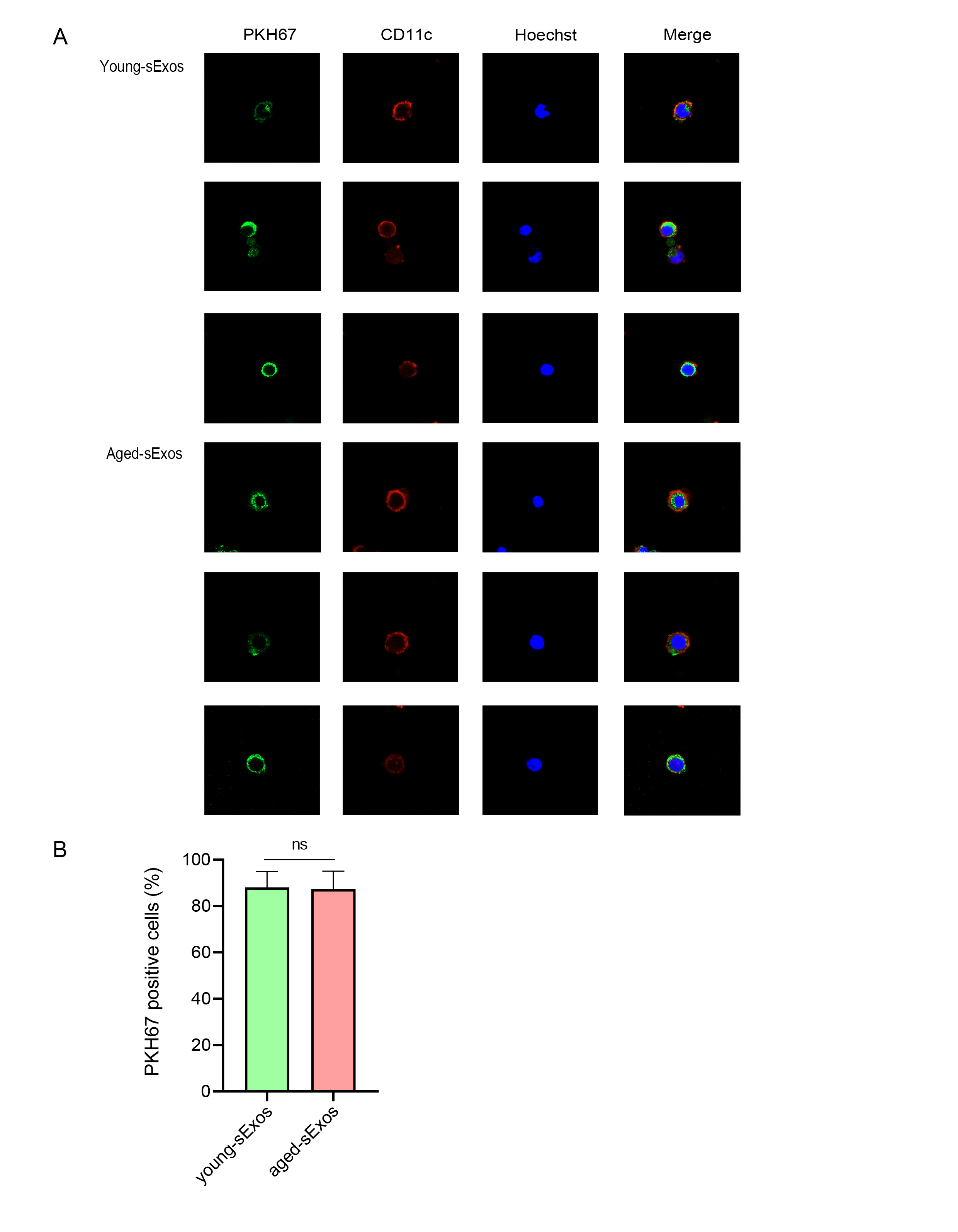

Supplement: Supplementary Figure 4 — Internalization of sExos by BMDCs Immunofluorescence staining of BMDCs after cultured with sExos from young male mice (A) and aged male mice (B). Red fluorescence shows antibody against CD11c, green fluorescence shows PKH67 labeled sExos and blue fluorescence shows nuclear (magnification, ×40). (C) Quantification of PKH67 positive CD11c+ cells. The differences between two groups were assessed by paired samples t-test, *P < 0.05 and **P < 0.01. (n=150). [file Image_4.tif]
